# Management of chronic non-communicable diseases in Ghana: a qualitative study of patients’ coping strategies and the role of caregivers

**DOI:** 10.1186/s12913-023-09398-4

**Published:** 2023-04-18

**Authors:** Robert Kokou Dowou, Hubert Amu, Farrukh Ishaque Saah, Lordina Ewurabena Arthur, Priscilla Aku Nuna Dotse, Luchuo Engelbert Bain

**Affiliations:** 1 Department of Epidemiology and Biostatistics, Fred N. Binka School of Public Health, University of Health and Allied Science, Hohoe, Ghana; 2 Department of Population and Behavioural Sciences, Fred N. Binka School of Public Health, University of Health and Allied Science, Hohoe, Ghana; 3 Department of Psychology, Faculty of Humanities, University of Johannesburg, Auckland Park, Johannesburg, South Africa; 4 International Development Research Centre, IDRC, Ottawa, Canada

**Keywords:** Chronic non-communicable diseases, Caregivers, Coping, Strategies, Management, Ghana

## Abstract

**Background:**

Chronic Non-Communicable Diseases (CNCDs) has become a major cause of mortality and disability globally. We explored the coping strategies adopted by CNCD patients and the roles of caregivers in the management of CNCDs in Ghana.

**Methods:**

This was a qualitative study that adopted an exploratory design. The study was carried out at the Volta Regional Hospital. Purposive convenience sampling procedures were used to sample patients and caregivers. Data for the study were collected using in-depth interview guides. Data were collected among 25 CNCDs patients and 8 caregivers and analysed thematically using ATLAS.ti.

**Results:**

Patients adopted a variety of strategies to cope with their condition. These strategies were emotion-oriented coping, task-oriented coping, and avoidance-oriented coping. Family members were the main caregivers, who provided social and financial support for patients. Financial challenges, inadequate family support, poor attitudes of health workers, delays at the health facility, unavailability of drugs at the facility, and patients’ non-adherence to the medical advice were major challenges that militated against caregivers’ efforts in supporting patients in the management of their CNCDs.

**Conclusion:**

We found that patients adopted various strategies to cope with their conditions. The roles of the caregivers in supporting patients in the management practices were identified as very important as they contribute immensely to the financial and social support for the patients in their management of CNCDs. It is crucial that health professionals actively involve caregivers in every aspect of the day-to-day management of CNCDs as these caregivers spend more time with these patients and understand them better.

## Introduction

Globally, Chronic Non-Communicable Diseases (CNCDs) have become the major cause of mortality and disability [1]. Globally, 41 million mortalities are recorded each year, equivalent to 71% of all deaths as a result of CNCDs (World Health Organization [WHO], 2018). CNCDs are driven by a myriad of factors that include rapid unplanned urbanization, globalization of unhealthy lifestyles, and population aging [1]. CNCDs are also known to be often associated with older age groups, but recent evidence shows that 15 million of the 41 million yearly deaths from CNCDs occur between the ages of 30 and 69 years [2].

Developing countries are disproportionately affected by the burden of CNCDs. For instance, over three-quarters of all CNCD mortalities (931.5 million) in 2016 occurred in low- and middle-income countries (LMICs) [3]. People in LMICs had the highest risk of dying from a CNCD (21% and 23%, respectively), nearly double the rate for adults in high-income countries (12%) [4].

Sub-Saharan Africa (SSA) is one major region of the developing world experiencing an exponential increase in the prevalence of CNCD and its associated mortality. Over the last two decades, the burden of CNCDs has surged in SSA and is projected to surpass communicable diseases as a major contributor to morbidity and mortality by 2035 [5]. Additionally, by 2030, approximately 46% of all mortality in SSA is expected to be attributed to CNCDs [6].

CNCDs management involves several practices that retain people living with these conditions as healthy as possible through the prevention, early detection, and management of CNCDs through the active involvement of the patients and the caregivers [7]. Besides the medical burden posed by CNCDs on the individuals, they also cause financial difficulties for the individuals and their families, because CNCDs are likely to ruin a family’s economic prospects [8].

Despite the importance of effective management of CNCDs, only a little attention has been given to it in the health system of Ghana. For instance, in the Ghana health system, there is inadequate rehabilitation facilities and the non-existence of homecare service for patients with CNCDs. Also, coping strategies adopted by patients in dealing with their conditions and the role the caregivers (relatives) play in the management of their relative’s condition (s), are inadequately recognised by health professionals. Living and dealing with CNCDs come with emotional and cognitive exhaustion since patients with CNCDs grapple with psychological depletion, and frustrating physical pain, hence, coping with these physical and emotional stresses by the patients is very important. We, therefore, explored the coping strategies adopted by CNCD patients and the roles of caregivers in the management of CNCDs in Ghana. We believe the findings from this study could serve as a source of reference for further studies on the management of CNCDs. Further, the findings could provide scientific evidence that may be tapped into by policymakers and program planners to empirically formulate policies and interventional programmes to improve the management of CNCDs and the involvement of caregivers in the management practices.

### Theoretical issues

**Fig. 1:**
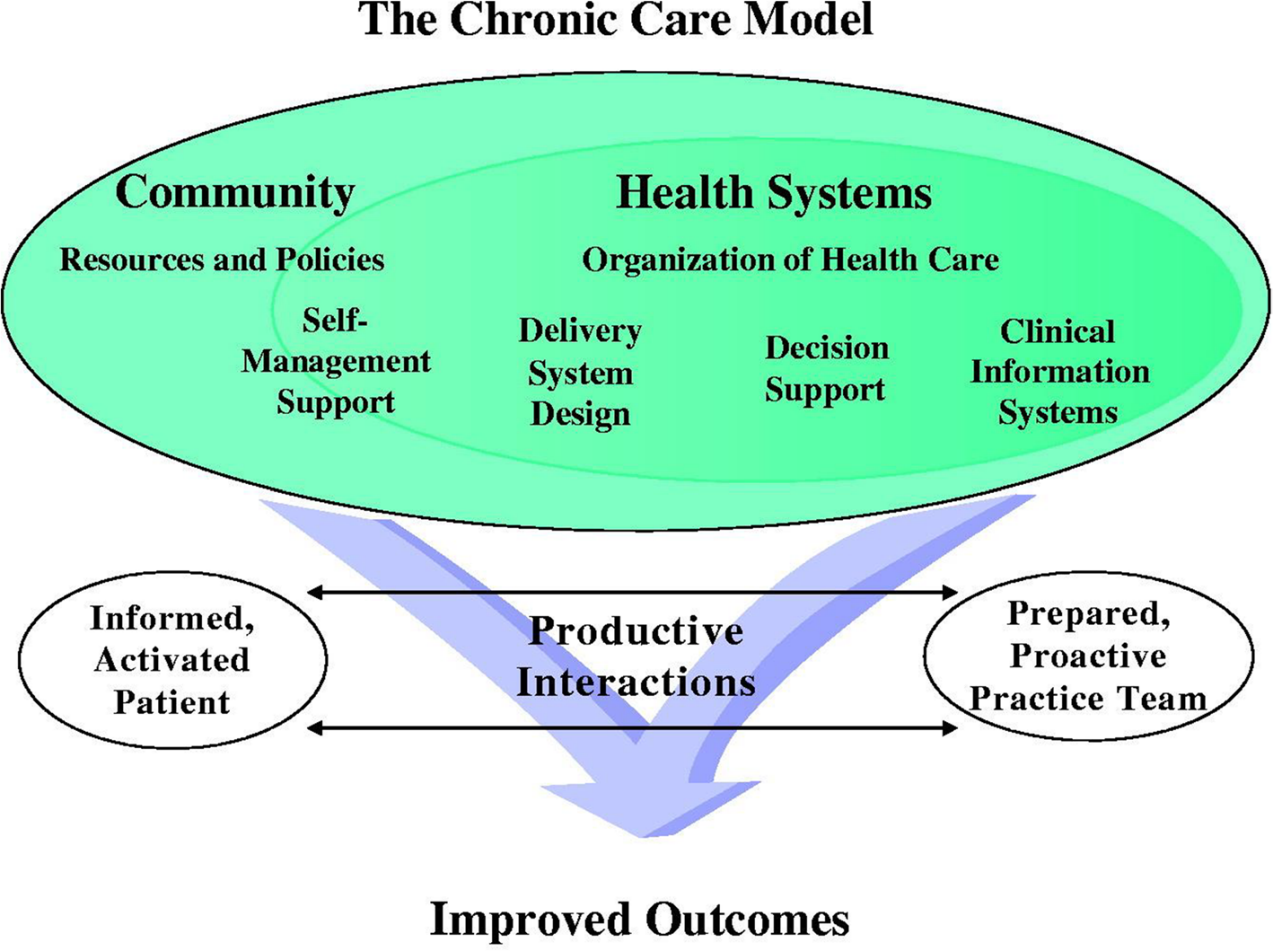
Conceptual framework: CHRONIC CARE MODEL

We reviewed the chronic care model (CCM) (Fig. 1) and the task-oriented, emotion-oriented, and avoidance-oriented coping framework to underpin our study. The CCM was developed by the MacColl Institute for Healthcare Innovation at Group Health Cooperative in 1992 [9, 10]. The CCM hypothesizes that improved health outcomes of persons suffering from CNCDs depend basically on indispensable components of a collaboration between the health care providers, community (caregivers), and patients [11]. The constructs encompass health systems, decision support, clinical information systems, patient self-management support, community resources, and delivery system design [12]. The Health Systems component describes the culture and strategies that create a corporate organization that provides safe, high-quality care to patients [12].

The delivery system construct describes the strategies and approaches needed to assure efficient, effective care, and self-management support for CNCD patients. This component also emphasizes regular, proactive planned visits which incorporate patient goals to help patients maintain optimal health and encourage patients to effectively cope with their conditions [13]. The patient self-management support construct theorizes that improved outcome for CNCDs management mainly depends on how individuals with CNCDs are prepared and empowered to take charge of their own healthcare outcome by fully participating in the management of their condition [9]. Another aspect of ensuring self-management support for patients is to provide self-management support to patients by mobilizing internal and community resources including the caregivers to provide ongoing self-management support to patients [13]. The tenets urge health institutions to form partnerships with informal caregivers in the management of patients [12].

The CCM has been considered relevant in this study because it is a well-established framework. This application of the theory had been in juxtaposition with the key findings of the study. The model facilitated exploring the role of patients in managing their health through the provision of self-management support strategies such as the setting of management goals, compliance with treatment and follow-ups, and coping mechanisms. The theory had been employed to explain how the community and caregivers support the management of patients’ conditions including the mobilizing of internal and community resources to provide ongoing self-management support to patients and address possible challenges that come with the management.

The task-oriented, emotion-oriented, and avoidance-oriented coping framework by Parker and Endler [14] has also been used in explaining how patients with CNCDs effectively adopt strategies to cope with their conditions. Parker and Endler [14] noted that many coping styles are adopted by individuals with stressful situations (CNCDs) in dealing with their stress. These include problem- vs. emotion-focused coping which indicated that problem-focused coping stratagems are associated with a task orientation, while emotion-focused ones reflect a person orientation.

Task orientation refers to strategies used to solve a problem, cognitively reconceptualize it, or minimize its effects [14]. On the other hand, person orientation denotes strategies adopted that include self-preoccupation, emotional responses, and fantasizing reactions [14]. Avoidance-oriented coping involves both task-oriented, and person-oriented strategies. Task-oriented avoidance is theorized as a distraction, while person-oriented avoidance takes the form of social diversion. A person may avoid a stressful situation (pain) by engaging in substitute or distractive activities (e.g., watching television or movies) or seeking out other people (social diversion). In task-oriented coping, the individual is confronting the stressful task. In distraction coping, the person is substituting an alternative task of his or her choosing that divert their attention from their current situation [14]. On the other hand, social diversion is “person-oriented in that the individual tries to “lose himself” by being with other persons rather than confronting the stressful situational task” [14].

## Methods

The reporting of this study is underpinned by the consolidated criteria for reporting qualitative research (COREQ) [15, 16].

### Setting

The study was conducted at the Volta Regional Hospital which was established in 1940 [17]. The hospital is located in Hohoe Municipality. Hohoe Municipality is one of the 18 Administrative Municipalities in the Volta Region. The population of the Municipality is 167,016. The Municipality has a total land area of 1,172 km2, which is 5.6 percent of the land area of the region. It is located in longitude 00 15’E and 00 45’E and latitude 6° 45’N and 7° 15’N. The majority (89.1%) of the residents of Hohoe are Christians, followed by Islam 7.8 percent; Traditionalists 1.2 percent and others religions less than one percent. The hospital is a moderately-sized public hospital with about 178 bed capacity serving the northern part of the Volta region of Ghana. The Hospital’s location, which is central to the Oti and Volta Regions makes it a major referral point especially from the Oti enclave and also from the neighboring country, Togo. The hospital is government-run and provides 24 h services which include laboratory and blood bank services, pharmacy services, x-ray and ultrasound scanning services, dental services, eye care and optical services, surgical services, and obstetric, gynecological, and maternal care services. The hospital also provides chronic care services to people with various CNCDs in specifically designed clinics.

### Study design

Our study was qualitative in nature and adopted an explanatory study design. In-depth interview guides were used in collecting data from the patients living with CNCDs including Hypertension, Diabetes, Glaucoma, Stroke, and Sickle cell seeking care at the facilities, and the caregivers who assist patients in their management. We employed this design because it helps in generating in-depth information about a specific issue of interest [18]. As such it helped this study explore comprehensively the coping strategies adopted by patients and the role the caregivers play in the management of the CNCDs.

### Study population

The main target population for our study was people living with CNCDs irrespective of the duration of living with the condition and the caregivers who assist these patients in the management of their condition (s). Inductively, the study recruited patients that were living with CNCDs that are managed in the hospital specifically those people with hypertension, diabetes, Glaucoma, stroke, and sickle cell. All the patients included in the study were outpatients who usually come to the hospital on regular days for their scheduled review.

### Sampling procedure and sample size

We used purposive and convenience sampling procedures for sampling participants. The purposive approach was employed to sample patients who were accessing treatment for CNCDs at various departments of the hospital (Physiotherapy department, Hypertension and Diabetes department, and Eye Clinics) and the caregivers that accompanied them to the facility. We sampled 25 patients living with CNCDs irrespective of the duration of living with the specific condition and 8 caregivers (relatives). The sampling size of patients was based on data saturation [19] and we reached saturation at the 25^th^ interview. However, the sampling of the caregivers was based on convenience, hence we selected only caregivers that were present with the patients at the time of data collection.

### Ethical considerations

Ethical clearance (Reference number: UHAS-REC A.9 [36] 20–21) was obtained from the University of Health and Allied Sciences Review Committee and authorization from the hospital before data was collected. Informed consent had been obtained from participants before including them in the study. This had been done by giving them informed consent forms to sign, indicating their willingness to participate in the study. The purpose of the study was also explained to them before interviewing them. They were informed of their right to stop at any point in time of the interview process when they feel so, and also assured that any statement or comments including that of privacy will remain protected. The confidentiality of the participants was adhered to by ensuring that no information from participants was disclosed to any third party. The respondents were recognized by codes and numbers instead of their real names. The computer which is used for the transcription of interviews was protected with passwords. To ensure the safety and protect the participants of this study from COVID-19, the necessary Covid-19 precaution had been taken by the research team including wearing masks, using hand sanitiser, and staying at least one meter away from the participants during the interviews.

#### Data collection methods

Data were collected face-to-face using in-depth interviews in collecting data from the patients and caregivers through face-to-face interactions. The majority of the patients’ and caregivers’ interviews were conducted in Ghanaian languages (Ewe and Twi). The choice of local language for the conduct of patients was to ensure that we have in-depth information without any language barrier challenges. To ensure that the use of the local languages did not affect the quality of the data collected, the research assistants that assisted in the collection of the data were appropriately trained before the data collection. The interviews were only conducted once and they lasted for 20–25 min.

#### Data collection instrument

Data for the study were collected using in-depth interview guides. The guide for patients focused on the socio-demographic and coping mechanism adopted by patients. Also, the guide for the caregivers was based on the socio-demographic characteristics of the caregivers, the various role they carried out in assisting patients in the management of their condition (s), and the challenges associated with the caregiving roles. Both an audio recorder and hand-written notes were used to record the interviews. The use of both the audio recorder and hand-written notes was to ensure that the interview process is not halted should any of the equipment (pen or audio recorder) breakdowns during the interview process. We recognise the importance of rigor and trustworthiness in qualitative research, we worked to ensure confirmability and transferability. The study findings were transferable and confirmable due to the detailed description of the study circumstances and techniques. After each interview, field notes were taken and referred to during the analysis, which included the participants’ nonverbal indications, worries, and interviewers’ reflections. Also, the instrument and the interview guides were given to experts in the field of qualitative study for them to peruse in detail the worth of the questions**.**

#### Data processing and analysis

All the recorded interviews from the participants were taken during the interview were transcribed into a word document from which codes and themes were developed using flexible thematic analysis (Braun & Clarke, 2019). We listened to the recorded tapes and using the field notes taken, the transcripts were read and edited to resolve any omissions and mistakes in the original transcripts. ATLAS.ti v7.5 was used in developing the codes and themes which were both a priority and emergent in nature. Familiarisation with the data was done to take note of key ideas and recurrent themes. Coding was done based on the research objectives as well as themes that emerged from the data itself. Quotes from the participants were used in presenting the data to substantiate issues discussed by participants. The themes were defined and named and a detailed analysis was conducted and written based on how they fit into the broader story of the data. To ensure the validity of the analysis, we used extracts from the data which capture the essence of each theme being demonstrated to develop the full write-up of the report. A frequency table was, however, used to present the socio-demographic characteristics of the Participants.

## Results

We present the results of this study based on the themes from the analysis conducted. The background characteristics of the participants are also presented.

### Background characteristics of study participants

Table 1 presents the background characteristics of patients. We noted that the majority of patients were 60 years and above (64.0%). Females constituted the majority (64.0%) of patients. The relative majority of the patients (48.0%) were married. The majority (52.0%) of the patients had SHS/O’level/A’level education while 8.0% had no formal education. Regarding occupation, the participants were generally farmers (28.0%), traders (40.0%), civil servants (8.0%), retired civil servants (12.0%), artisans (4.0%), drivers (4.0%), and students (4.0%). We noted that 32.0% of patients were having comorbidities. Hypertension was present in almost all the co-morbid cases recorded. Approximately, 32.0% of the patients had lived with their respective condition(s) for 6–10 years.

**Table 1:** Socio demographic characteristics of patients

Variables	Frequency (%)	Percentage (%)
Age		
< 30	1	4.0
30–39	1	4.0
40–49	2	8.0
50–59	5	20.0
60 +	16	64.0
Sex		
Female	16	64.0
Male	9	36.0
Ethnicity		
Ewe	12	48.0
Akan	4	16.0
Guan	5	20.0
Muslim	4	16.0
Marital status		
Never married	2	8.0
Married	12	48.0
Divorced	3	12.0
Widowed	8	32.0
Level education		
None	2	8.0
Primary	4	16.0
SHS	13	52.0
Tertiary	6	24.0
Religion		
Christianity	22	88.0
Islam	3	12.0
Occupation		
Student	1	4.0
Civil servant	2	8.0
Retired civil servant	3	12.0
Farmer	7	28.0
Trader	10	40.0
Driver	1	4.0
Artisan	1	4.0
Place of residence		
Hohoe	17	68.0
Jasikan	8	32.0
Condition		
Hypertension	9	36.0
Stroke	5	20.0
Diabetes	2	8.0
Sickle cell	1	4.0
Glaucoma and Hypertension	1	4.0
Hypertension and Diabetes	5	20.0
Hypertension and Stroke	2	8.0
Duration of living with condition (In years)		
< 1	3	12.0
1–5	7	28.0
6–10	8	32.0
11–15	3	12.0
16–20	3	12.0
21 +	1	4.0
Duration of attending facility		
< 1	2	8.0
1–5	12	48.0
6–10	5	20.0
11–15	4	16.0
16–20	2	8.0
**Total**	**25**	**100**

Table 2 presents the socio-demographic characteristics of caregivers. The majority of the caregivers (50.0%) were in their 40 s and Ewes in terms of ethnicity respectively. The majority of the caregivers (62.5%) were married. All the caregivers were Female and Christian. The majority (50.0%) of them had JHS level education whiles 25.0 percent had a tertiary level of education. Regarding occupation, the participants were generally traders (75.0%), farmers (12.5%), and retired civil servants (12.5%). The majority (87.5%) of patients were residing in Hohoe Municipality (Volta region) while 12.5 percent of them were from Jasikan (Oti Region).

**Table 2:** Socio-demographic of caregivers

Variables	Frequency	Percentage
Age		
< 30	1	12.5
30–39	1	12.5
40–49	4	50.0
50–59	1	12.5
60 +	1	12.5
Sex		
Female	8	100
Ethnicity		
Ewe	4	50.0
Akan	3	37.5
Guan	1	12.5
Marital status		
Never married	2	25.0
Married	5	62.5
Divorced	1	12.5
Level education		
JHS	4	50.0
SHS	2	25.0
Tertiary	2	25.0
Religion		
Christian	8	100
Occupation		
Farmer	1	12.5
Retired teacher	1	12.5
Trader	6	75.0
Place of residence		
Hohoe	7	87.5
Jasikan	1	12.5
Total	8	100

### Thematic results

#### Coping strategies of patients

Table 3 presents the main and sub-themes that emerged from the interviews conducted among the patients. Three main themes were realised: the main coping strategy adopted by the patients in dealing with their conditions; the source of the coping strategy; and the level of success of the strategy adopted in dealing with the diseases.

**Table 3:** Coping strategies adopted by patients in dealing with their diseases

Main Theme	Sub-theme
**Main strategy adopted**	**EMOTION-ORIENTED**
	❖ **Religious activities**
	✓ Prayer
	✓ Faith,
	✓ fasting,
	✓ Going to church
	✓ Sing and dance,
	✓ Read bible
	❖ **Creative art**
	✓ Listening to radio (gospel music)
	✓ watching television
	**TASK-ORIENTED**
	❖ **Engaging in social activities**
	✓ Visiting friends,
	✓ Chatting with friends,
	✓ Playing games,
	✓ Playing football with friends;
	✓ Going out to stand at the road side,
	✓ Going around the town (roaming);
	✓ Doing exercise
	**AVOIDANCE-ORIENTED**
	❖ **Intrinsic Adaptation**
	✓ Try and make my mind not to be thinking of the conditions and my problems
	✓ Take the condition as other conditions
**Source of coping strategy**	**Self**
	✓ A personal decision not to worry or think about the condition nor the challenges
	**Family and friends**
	✓ Spouse, parents, children
	✓ Close friends (lecturer
	**Health professionals**
	✓ Nurses advise
	✓ Doctors
	**Religious means**
	✓ Bible,
	✓ pastors
**Effectiveness of coping mechanism (Level of success with strategy)**	✓ Improvement in the condition
	✓ prevented the deterioration (stable Bp status)
	✓ become resilient (don’t think of the condition, less anxious and relaxed)

Concerning the main coping strategy adopted by the patients in dealing with their conditions, three sub-themes emerged. These were; Emotion- oriented (religious activities and creative art); Task-oriented (social activities and physical activities) and Avoidance-oriented (intrinsic Adaptation).

Apropos the emotion-oriented coping, religious activities including prayer, having faith in God, fasting, and going to church and the use of creative arts such as listening to the radio or watching television were emotion-oriented coping strategies reported by the patients in their interviews.

Regarding religious activities, having faith in God, fasting and prayer were the main strategies adopted by most of the patients in dealing with their conditions. To patients, praying to God either themselves or through their pastors, fasting, and strengthening their faith in God, help them through the predicaments of living with chronic non-communicable diseases with fewer worries. Other patients also indicated that going to church regularly helps them to deal with loneliness which can make them think about the conditions they are inflicted with.

The majority of patients held the belief that God has the supreme power that could heal their diseases if they fast and pray. The majority of the patients further opined that when they pray, they get encouraged to keep living normally, as though they did not have chronic diseases. The following quotes, summarise the views of the patients:

*I go to church every time to pray to God to help me go through this condition. When our pastor preaches to us in the church, it fortifies me and gives me hope that God will surely heal me, because he does everything and everything is in his hands.*

–Diabetes and hypertension patient, Male, 62 years

*It is the prayer, you have to pray to God to help you and take the condition from you, so I have been praying, because God is the healer, so I have faith that I will be cured one day because no one can cure you apart from God.*

–Diabetes and hypertension patient, Female, 67 years

Relating to using forms of creative arts as a strategy for coping with chronic non-communicable diseases, the relative majority of the patients revealed that they can cope with their ailments by watching television, movies, and or listening to the radio. They explained to enjoy entertainment (football), and church programmes on tv and radio stations. These strategies according to them, serve are distracting approaches that make them forget about their diseases briefly. Thus, they make them not to be thinking about the fact that they are living with a chronic non-communicable disease. The following quotes summarise their views in this regard:

*I watch Television a lot, and because of that, I bought DSTV and watch football. If you are watching it (football) your mind will be on it so you will not think much about your condition.*

–Stroke patient, Female, 60 years

*Yes, I watch movies, sometimes I can be watching them till I fall asleep, the Ghanaian movies are funning so when you are watching them, you will laugh and you forget about your problems.*

–Hypertension patient, Female, 65 years

To limit the impact of the emotional and psychological stress that was associated with living with CNCDs, some of the patients indicated that they engage in some social activities that help them to cope (Task-Oriented Coping). These tasks include visiting friends, exercising, going out to roam in town, going to stay at the roadside, church meetings, and playing games and football with friends. These strategies, according to the patients, help them to forget the fact that they are living with their respective diseases. The following quotes summarise their responses:

*I like playing cards and “Ludo” with my friends, previously I used to play football. However, when I realized it was not good for me, I stopped, though I play sometimes I will just play small for about five minutes and I stop. Playing game with my friends take my mind away from my condition totally.*

–Sickle Cell patient, Male, 18 years

*If my friends and brothers are in the house and we are chatting or playing cards, I forget about my condition and feel better. But if they are not around and I am not doing anything, I play “Ludo” alone or play games on my phone.*

–Diabetes and hypertension patient, male, 54 years

It was realized from the response of patients that some of them relied on self-motivation by psyching themselves not to think or worry over the condition by just accepting their condition (s) as one of the other physiological illnesses (Avoidance-oriented coping). They explained that thinking about the condition always only aggravates their health statuses hence shifting their mind off their circumstances. The following quotes summarise their responses:

*I don’t stress myself, whatever they asked me to do I do it. I always wear my jacket, I don’t go to places where there is too much crowd so I am just in my comfort zone, and if I don’t have anything to do, I just sleep.*

–Sickle Cell patient, Male, 18 years

*You see when it (condition) happened, I just took it as one of those diseases that affect everybody, so I turn my mind away from the condition so I will be okay, and I have realized that I am improving fast, so I do not think and I have faith that one day it (condition) will go.*

–Stroke patient, Female, 60 years

### Source of coping strategies adopted by patients in dealing with their condition

The sub-themes which emerged based on sources of coping strategies adopted by the patients were; self; friends; health professionals; and religious faith.

It is also worth indicating that some of the patients explained to have learned their coping mechanisms from many sources including their initiatives. For instance, a 65-years- old, Female hypertension patient noted: “*The nurses tell us some, as for the prayer and church it was from me. My pastor also told me not to think much about the condition because that will increase my BP*”.

With the regard to the sources of the various coping strategies patients adopted in dealing with their respective chronic non-communicable diseases, the majority of the patients indicated that the various mechanisms they have adopted emanated from their ingenuity. The following quotes summarise their responses:

*I came about all the coping strategies myself so that I do not worry about my current predicament. When I try one strategy and it did not work for me, I try a new one, till I get the right one that could help me live my life and get better quickly.*

–Stroke patient, Male, 60 years

*I developed an interest in card playing so that I can entertain myself and I made my friends help me learn how to play it well, so I have the idea of the game but it was my friend who thought me how to play it.*

–Sickle Cell patient, Male, 18 years

For some of the patients, the source of coping they had adopted had to do with family and friends. To them¸ it is their friends and family members who motivate them to be strong and live positively with their respective diseases. It is through their advice and encouragement that the patients were able to effectively cope with their diseases. Some of them had these to say:

*A lecturer who is my best friend once told me that I should not stress myself too much, I should not be thinking a lot because some people get it (condition) before me and some of them have completely healed and they go about their activities, so sometimes I do confront them for their advice.*

–Stroke patient, Male, 46 years

*It was a friend who told me to try the strategy. I have tried it and it is working for me. It was not that easy at the beginning but when we come to the review, he will encourage me to keep on trying it. He said that I will get used to it and indeed, I am doing it and it is helping.*

–Diabetes and Hypertension patient, Male, 62 years

Some of the patients also noted that the strategies they adopted in coping with their diseases were derived from the health professionals such as the doctors and nurses who manage their conditions, they explained that the strategies adopted mainly originated from the advice, and education that the health professionals give them regarding their conditions and how they are to be managed.

Some of them had these to say in that regard:

*Yes, I also learn most of the things I do to cope from the counselling session. When we go to the hospital, they (nurses) told us not to think much, because it is not good, and when you are thinking the medicine will not work. So from that advice, I just try to keep my mind away from my condition and live my life in a way that can improve my condition.*

–Hypertension patient, Female, 53 years

*The health professionals have been teaching us here (hospital) when we come for review. Their teaching has helped me in many ways, so what I do now to relax and not worry about this devil condition comes from the nurse that takes care of us. They are friendly, so they talk to us about everything we need to manage our condition.*

–Diabetes patient, Male, 63 years

The majority of patients who indicated that prayer and belief in God were their main strategies, noted that they adopted these strategies based on their religious faith. To many of them, God is the source of their ability to cope with their respective conditions. Aside from God, pastors also served as an important source of coping for them, since they encourage them to put their faith in God and remain strong despite living with the diseases. The Bible according to those who were Christians, also constituted an important source of motivation for them. When they read the bible, it enables them not to worry about their circumstances as it offers the strength and hope to carry on. The following quotes represent some of their responses:

*Since childhood, I go to church with my parents, because my parent were Christians. So I got everything from the bible. Also when I go to church, the pastor preaches and the word of God motivates me to keep going.*

–Hypertension patient, Female, 53 years

*I learned it from the church through the pastor’s preaching and I read it in the holy textbooks (Bible) also that with God all things are possible, life and Dead is in the hand of our Lord Jesus Christ, so when I believe in him, I know one day it (condition) will come down.*

–Diabetes and Hypertension patient, Male, 64 years

### Effectiveness of the coping mechanisms

Vis-à-vis the usefulness of the adopted coping strategies in dealing with chronic non-communicable diseases, a few of the patients have revealed that some of the coping mechanisms they have adopted have helped in one way or other in ameliorating their condition. The following quote illustrates their responses:

*Previously, I cannot even walk from here to there but since I have started going to church and also stop thinking too much and taking my drug, I can even now walk from here to Kpoeta (a nearby community).*

–Hypertension patient, Female, 53 years.

*Yes, previously, this arm cannot move at all but now it is working and moving. So it has helped me improve my condition because I am not thinking as I used to do and I make sure I also do what the doctors tell me. So all of them are helping me to get better.*

–Stroke patient, Female, 72 years.

### Role of caregivers in the management of CNCDs

Table 4 presents themes derived from the interviews conducted among caregivers. From the caregivers’ interviews, six main themes emerged. These were; centrality in the management, support of caregivers in the management, the effectiveness of the support, the challenges associated with the support, and suggestions to address challenges associated with the support.

**Table 4:** Role of caregivers in the management of chronic non-communicable diseases

Main theme	Sub-theme
**Role of Caregivers** (Family, friends, and house help)	**Social support**
	✓ Encourage and help to exercise
	✓ help take medications on time
	✓ Cooking and feeding patient
	✓ Washing (patient hygiene)
	✓ review
**Effectiveness of the supports**	✓ improvement in the condition
	✓ adhere more the drugs and exercise
	✓ fast recovery
**Challenges**	**Financial challenges**
	✓ for medication
	✓ surgery
	✓ transportation for review
	**lack of the family support**
	Attitude of health professionals
	delay at the facility (waiting time)
	patient resisting to adhere to the advice

Caregivers are intrinsic roles in the management of chronic non-communicable diseases of their relatives (patients). Despite that one caregiver indicated being hired as a caregiver, the rest of the caregivers were family members. The key family members who served as the caregivers were; the parents, siblings, spouses, and children of the patients. Extended family relations (relatives) such as nieces, and in-laws also served as caregivers to a number of the patients.

Concerning the social supports given by the caregivers to the patient in the management of their CNCDs, the caregivers indicated that their social supports entailed cooking and ensuring that patients eat on time, ensuring that patients take medications on time, ensuring the hygiene of patients (washing and bathing), encouraging and supporting patients to exercise; accompanying and staying with the patient at the hospital (review), monitoring the patient while in the house (safety), and psychological and emotional support (counselling and motivation, advice patient not to worry too much about the condition, sing for the patient).

Regarding ensuring that patients eat, take medication on time, and come to the hospital for reviews as scheduled, the caregivers for instance indicated that due to the nature of the condition of their relatives which made them unable to do anything for themselves, they have to practically do everything for them including cooking and ensuring that they eat and take their medication on time and also bring them to the hospital for reviews when the scheduled day is due. The following quotes summarised some of their responses:

*As for now, she (the patient) is blind so I do everything she needs for her. I bring her to the hospital, cook, if she wants to go to the washroom I take her there, I bathe her, give her medicine and sometimes if she wants to go somewhere I take her there.*

–Caregiver, Female, 39 years

*Because of her condition, I cannot allow her to go to the hospital alone, so when her review times are due at the hospital, I bring her, and I stay with her there till the health professionals have taken care of her.*

– Caregiver, Female, 50 years

Concerning emotional and psychological support, for instance, the caregivers noted that usually, due to the emotionally stressful nature of the conditions, their family members become depressed and wept a lot. They were then always available to support them emotionally and psychologically by encouraging, and comforting them: The following quotes are representative of the emotional support provided by caregivers to the patients.

*At times, she can get angry, without any reason. At times, she used to cry the whole day. Like yesterday like this, she asked me to sing for her, so, the whole of yesterday, I sang for her. When you stop, then she will start crying, so I have to be singing for her. Sometimes, I invite my friends to come to the house to help me to sing for her.*

– Caregiver, Female, 42 years

Yes, in the house, I always tell her not to think much because God knows why he has made her the way is now, so she should have faith in God and God will take care of her.

– Caregiver, Female, 39 years

Most of the caregivers explained in their interviews that they do sometimes provide patients with financial support to the patients. These supports according to them entailed payment of bills (lab, monthly review fees, and consultation), medications, and taking transportation to the health facility. The following quotes summarize these codes:

*Sometimes, she is an old person and has retired, so I have to help her financially by paying some of the hospital bills and buying some prescribed medications. Because of her condition, she has spent her savings, so I can’t sit and see her die like that, so I have to do anything possible to help her even though I have used all my savings.*

– Caregiver, Female, 50 years

*When her condition started, her fiancé and one of my family relatives used to support her financially, but, it has gotten to a time they got fed up and stopped. It is only I who is supporting her financially. I sell in the market, and the money I get, we use that money to come to the reviews at physiotherapy. I want her to get well fast so I use my money to do everything for her.*

– Caregiver, Female, 40 years

Regarding how effective the roles of caregivers are in the management of patients’ conditions, the caregivers in their responses to the interview explained that their support to patients has contributed to the improvement of the status of their relatives. The following quotes summarize the responses of some caregivers:

*Yes, I can say that the way her condition was at the begging, it has improved because I am with her always and taking care of her so if we need something like money, I give it out and she is not thinking anymore much like she previously do. Her condition is now better than previously.*

– Caregiver, Female, 47 years

*Please yes, because previously she was alone and I was at my husband's place and I have seen that she was suffering and she will be struggling to do everything alone, so I have decided to come to be with her for some time and I have been helping her with everything since. I have seen that she is better and not sick as was before.*

– Caregiver, Female, 42 years

### Challenges faced by caregivers in supporting patients in the management of cncds

Concerning the challenges that the caregivers face in supporting the patients in the management of their conditions, the caregivers reported that they face many challenges including Financial, challenges (for bills, medications, transportation, and surgery), inadequate family support; poor attitude of workers; delay at the facility (waiting time); drugs not available at the facility; patient resisting to adhere to the advice.

Regarding financial challenges, the caregivers noted that due to the financial burden of the management of CNCDs, the patients have spent most of their financial resources, hence they have to take over sometimes by paying for management costs and transportation to the hospital and these constitute challenges for them they do not even have much time to engage in any financial that can bring in revenue: The following quotes summarised their responses in that regard:

*Money is the main challenge we face. It has been a year since we have come here, Because there is no money for her to come to the hospital, we do miss review schedules sometimes. When you come to the hospital, they (health professionals) will write medicine for you and you need to buy them but there is no money to buy them. We have spent a lot of money since she having this condition.*

– Caregiver, Female, 39 years

*Even now we have to buy some medications. A nurse here asked us to buy them (medications) because her legs are getting swollen. But I told her I can’t afford them because I don’t have no money. When I go outside to look for money, I don’t know what might happen to her in the house. Maybe she might feel like going to private or something. She can’t talk so I decide not to go to any place at all.*

– Caregiver, Female, 39 years

Inadequate family support from other family members was also noted by caregivers as one major challenge they face in their efforts to support the patients. They noted that since they have stepped in to care for their relatives, other family members have not supported or provided necessary assistance, therefore, leaving all burden on them and this made them become very overburdened and stressed. Some of their views are summarized in the following quotes:

*For the children, they help, but other relatives like extended family members do not help us at all. Everything is left on my shoulder. sometimes, I become very tired. But I did not have any other option than to continue helping her.*

– Caregiver, Female, 47 years

*Other relatives are not helping, I will come to the hospital, help her take medicine, cook for her, and at the same time have to go and cook for my husband, so they need to help me, me alone I am tired. I do become very stressed, moving from my husband’s place to her place and at the same time going to market is not easy. If I have someone that can assist me, I can also rest small.*

– Caregiver, Female, 26 years

Poor attitude of health professionals; delays at the facility (waiting time); and patients’ resistance to adhere to advice were other challenges that were explained by the caregivers. Regarding the long waiting time at the facility, for instance, the caregivers opined that due to staff and the large number of patients to be served at a time they have to wait for long hours before they are attended to: The following quotes represent their responses on this challenge:

*The only challenge I face in the hospital here is the delay at the dispensary. You have to wait for a very long time and if you try to complain to them, they (Health professionals) will talk to you anyhow.*

– Caregiver, Female, 50 years

*You have to wait for long hours before they (Health professionals) take care of her. We can come here very early in the morning but since the doctor is the only one in the clinic, we have to wait for long hours before he calls us.*

– Caregiver, Female, 42 years

Concerning the reluctance of the relatives to adhere to the directives by the health, the caregivers revealed that their relatives were usually reluctant to adhere to self-management directives. On this challenge**,** a Female, 40 years caregiver stated this:

*She needs to stop taking some diets. But the one that she is not to take is the very one that she feels like taking. So, when I give her the other foods, she looks at it like I am punishing her or I don’t want her to do what she wants. Then sometimes she doesn’t want to listen to me. Because she finds it difficult to talk, she will put the food down without eating. At times too, maybe she is tired of doing exercise but I know that the more exercise she does, the more she gets better. But she will refuse to do it.*

*–* Caregiver, Female, 40 years

## Discussion

We found that patients adopt varieties of mechanisms to cope with their condition. These strategies were emotion-oriented coping, task-oriented coping, and avoidance-oriented coping. Prayer and engagement in social activities were key strategies patients adopted in coping with their conditions. Families were the main caregivers, who provided social and financial support for patients. Financial, challenges (for bills, medications, transportation, and surgery), inadequate family support; poor attitude of workers; delay at the facility (waiting time); drugs not available at the facility, and patient resistance to adhere to the advice were noted some challenges faced by caregivers in assisting patients in the management of their condition.

The result revealed that the main strategy adopted by most of the patients in dealing with their conditions were; emotion-oriented, task-oriented, and avoidance-oriented [14, 20]. For the majority of the patients, the main sources of their coping strategies had to do with themselves, friends, and in some cases health professionals. Coping is posited by the conceptual framework, as a way by which patients adapt to their chronic conditions and live as ordinary individuals in the community [10].

It was realised that the major emotional-oriented coping that is adopted by almost all the patients were prayer, fasting, going to church s, and the belief in God. The use of prayer and faith in God as a way to cope with stressful difficulties that come with living and managing CNCDs as observed in this current study corroborates with previous studies that have identified religiosity as a major coping mechanism among patients [21–26]. The predominant reliance on God as a coping strategy by people living with CNCDs in this study is not surprising because religions form an important aspect of Ghanaian culture and tradition. In addition, the observation made in the study could be due to the fact that Christianity constitutes the major religion in Ghana. As result, the majority of patients have faith in God and that He is capable of healing them [21, 24, 27]. An additional emotional-oriented coping strategy adopted by the patient was the use of creative arts such as listening to the radio or watching television. These creative arts help the patient to convalesce from emotional and cognitive exhaustion since patients with CNCDs grapple with psychological depletion, and frustrating physical pain [28–30]. This observation points to the role creative art plays in health promotion among people living with chronic diseases [3, 29, 31–33].

It was also realised in the current study that patients also adopt task-oriented strategies including visiting friends, exercising, going out to roam in town, going to stay at the roadside, church meetings, and playing games and football with friends as a coping strategy to limit the impact of the emotional stress that is associated with leaving with chronic non-communicable diseases [34]. This finding is consistent with the conceptual framework that emphasized the roles of the community including friends and neighborhoods play in patients’’ management of CNCDs [12]. This finding could be explained by the fact that people living with chronic conditions engage or resorted to social activities as a way to avoid thinking about their conditions [20, 35]. This observation in the present study, where patients engaged in social activities such as visiting families and friends as well as attending social gathering as a coping mechanism point to the role of families and friends in enabling patients with the chronic non-communicable disease to cope with their conditions. This is consistent with the findings of previous studies where patients suffering from chronic diseases sought social support by talking to families and friends as a way of coping with their conditions [25, 36–39].

Furthermore, the study revealed that some of the patients dealing with their condition adopt an avoidance-oriented coping strategy that involves patients self-motivating or psyching themselves not to think or worry over the condition by just accepting the CNCDs as one of the other physiological illnesses [40, 41]. This avoidance coping mechanism among patients could be due to the fact many of them believe that overthinking over the condition always only aggravates their health status hence shifting their minds off their circumstances could help stabilise their health status [20]. This belief makes them more resilient to all challenges that come with living with CNCDs [42]. This intrinsic adaptation, stress appraisal, and self-motivation as a coping strategy by patients are consistent with the findings from previous studies [35, 43].

Vis-à-vis the usefulness and effectiveness of the various coping strategies adopted, it was noted that most of them indicated that the strategies they have adopted were successful in the management of their respective conditions and this is congruent to previous studies which found coping strategies adopted by patients living with chronic diseases as very successful [44–46].

Caregivers play fundamental roles in the management of chronic non-communicable diseases of the patient. They perform varieties of roles to help the patient deal with the social, psychological, physical, and economic challenges that are associated with living with CNCDs. It was realised that the major functions the caregivers play include providing social support such as ensuring eating and taking of medication on time, encouraging and supporting patients to exercise, accompanying and staying with patients at the facility, and financial support such as paying medical bills, surgeries and transportation for reviews. The role of caregivers was imperative because most of the patients living with NCDs are old aged and physically and financially incapacitated as result, they are unable to do much personal care and house chores for themselves. Also, due to the CNCDs, most of the patients are unable to engage in any financial renumerating employment while the management of their condition is financially demanding [47].

These roles were perceived by caregivers to have been effective in improving the condition (s) of their relatives. In Ghana, major caregivers that assist patients in the management of their condition (s) are families. This observation is in line with the conceptual framework of the present study that argues that community support patients including social, financial, and emotional support from various aspects of the community are very imperative in helping patients in dealing with the predicaments that are associated with living with the CNCDs [12]. These findings also point to the central place family holds in the existence and living of individuals in Ghanaian society. The findings in this study where family plays a major role in the health care of patients are consistent with previous studies [47–50].

### Strength and limitations

The current study was a health facility-based study designed to have an in-depth understanding of the management of CNCDs from the perspective of patients and caregivers in the Volta Region. The qualitative nature of the study made it easier to unearth in detail issues regarding coping by patients and the role informal caregivers play in the management of patients’ conditions.

The use of in-depth interviews for the data collection could have introduced response bias on the part of the participants. The use of purposive sampling introduced the possibility of selection bias on the part of the data collectors. The possible implication of these biases is that all issues regarding the management of CNCDs might not have been entirely covered in the current study.

## Conclusion

We found in our current study that patients adopt varieties of strategies to cope with their CNCDs These strategies are emotion-oriented, task-oriented, and avoidance-oriented. Caregivers also play essential roles in supporting patients in the management of CNCDs. Our findings imply that for Ghana to achieve the 3.4 targets of reducing by one-third premature mortality from CNCDs through prevention and treatment by 2030, it is crucial that health professionals actively involve caregivers in every aspect of the day-to-day management of CNCDs as these caregivers spend more time with these patients and understand them better. The health system challenges that were realised in the study including the non-availability of essential drugs at the facility or the non-coverage of some of the chronic management costs by the national health insurance could prevent people from patients from seeking chronic care on time hence leading to complications and premature deaths.

## Recommendation

Our findings made it imperative to make some of the following recommendations.

Ministry of Health, the Ghana Health Service should ensure that essential drugs necessary for the management of CNCDs are available at the facility. National Health Insurance Authority together with other stakeholders should restructure the current National Health Insurance Policy to cover the management of all CNCDs and also cover all management costs including medication. Further, it is crucial that health professionals actively involve caregivers in every aspect of the day-to-day management of CNCDs since these caregivers spend more time with these patients at home.

## Data Availability

All relevant data are within the manuscript. Any further requests regarding the data used for this study could be made through the corresponding author.
